# Mechanism of Mechanical Trauma-Induced Extracellular Matrix Remodeling of Fibroblasts in Association with Nrf2/ARE Signaling Suppression Mediating TGF-*β*1/Smad3 Signaling Inhibition

**DOI:** 10.1155/2017/8524353

**Published:** 2017-10-03

**Authors:** Jianming Tang, Bingshu Li, Cheng Liu, Yang Li, Qiannan Li, Linlin Wang, Jie Min, Ming Hu, Shasha Hong, Li Hong

**Affiliations:** Department of Gynecology and Obstetrics, Renmin Hospital of Wuhan University, Wuhan, Hubei Province, China

## Abstract

Stress urinary incontinence (SUI) is a common hygienic problem affecting the quality of women's life worldwide. In this research, we revealed the involvement and regulation of extracellular matrix (ECM) remodeling, oxidative damage, and TGF-*β*1 signaling in the pathological mechanisms of mechanical trauma-induced SUI. We found that excessive mechanical strain significantly increased apoptosis rate, decreased cell viability and ECM production, and broke the balance of MMPs/TIMPs compared with the nonstrain control (NC) group. The expression levels of TGF*β*1, p-Smad3, Nrf2, GPx1, and CAT were downregulated, the production of ROS, 8-OHdG, 4-HNE, and MDA was increased, and the nuclear translocation of Smad2/3 was suppressed after 5333 *μ*strain's treatment. Both mTGF-*β*1 pretreatment and Nrf2 overexpression could reverse mechanical injury-induced TGF*β*1/Smad3 signaling inhibition and ECM remodeling, whereas mTGF-*β*1 had no effect on Nrf2 expression. Nrf2 overexpression significantly alleviated mechanical injury-induced ROS accumulation and oxidative damage; in contrast, Nrf2 silencing exhibited opposite effects. Besides, vaginal distention- (VD-) induced in vivo SUI model was used to confirm the in vitro results; Nrf2 knockout aggravates mechanical trauma-induced LPP reduction, ECM remodeling, oxidative damage, and TGF-*β*1/Smad3 suppression in mice. Therefore, we deduce that mechanical injury-induced ECM remodeling might be associated with Nrf2/ARE signaling suppression mediating TGF-*β*1/Smad3 signaling inhibition. This might reflect a new molecular target for SUI researches.

## 1. Introduction

SUI is a common social and hygienic problem affecting the quality of life of women worldwide and causing serious social economic load [1, 2]. Even though progress has been made in the surgical therapy of SUI in recent decades, the pathogenesis and pharmacological treatment for SUI are poorly understood. The most recognized theory of the pathogenesis of SUI is the integral theory, which indicated that mechanical trauma-induced ligament and nerve injury, as well as the damage to the vaginal wall and its suspensory structures, resulting from vaginal delivery is one of the widely recognized risk factors of SUI genesis in women [3, 4]. The vesical neck and urethra are attached to the anterior vaginal wall, which has fascial connections to the levator ani muscles through the arcus tendineus fasciae pelvis. In addition, connective tissues and muscular tissues enable the vaginal wall to have enough strength and toughness to maintain the normal anatomic position and function [3–5]. Mechanical injury of the pelvic floor (such as childbirth) may disrupt these supportive tissues and connections via the remodeling of ECM. Then, the urethra will lose the hammock-like support and result in SUI. With regard to the metabolism of ECM, some studies showed an increase of matrix synthesis in response to suitable mechanical strain [6, 7], while excessive force would reduce the production of ECM [8], which may be one of the main pathological mechanism of childbirth trauma-induced SUI.

As an important regulatory factor of ECM metabolism, TGF-*β*1 and its canonical downstream Smad proteins have been implicated in the regulation of mechanical trauma-induced SUI and many other ECM remodeling-related diseases, such as pulmonary fibrosis, myocardial hypertrophy, and angiosclerosis [9–11]. A suitable mechanical strain would activate the latent TGF-*β*1 and increase the production of ECM [10, 12], whereas the ECM production of pelvic tissues of patients with pelvic floor dysfunction or SUI mice models was significantly reduced [13–15].

ROS accumulation is a result of mechanical stress. A suitable mechanical strain can induce a physiological raise of ROS levels, while an excessive mechanical stress can cause an excessive ROS accumulation and result in oxidative damage [11, 16, 17]. Oxidative damage was reported to have been involved in mechanical trauma-induced pelvic floor dysfunction [18–20]. Nrf2 is a well-characterized, global antioxidant gene inducer, whose activity is tightly controlled by cytoplasmatic association with its inhibitor Keap1. Upon oxidative stress, Nrf2 dissociates from Keap1, translocates into the nucleus, and transactivates antioxidant genes, such as GPx1, MnSOD, CAT, HO-1, and so on [21].

The dynamic equilibrium of MMPs and TIMPs is a critical factor of ECM metabolism and was confirmed to have been involved in mechanical injury-induced pelvic floor dysfunction and mechanical stretch-induced elasticity change of the blood vessel wall [14, 22, 23]. Besides, both oxidative stress and TGF-*β*1 may regulate the expressions and activities of MMPs and TIMPs [14, 22, 23].

Based on the above thesis, we hypothesized that the oxidative stress and TGF-*β*1/Smad signaling may be involved in mechanical trauma-induced SUI. In the present study, we explored the involvement of oxidative stress-related Nrf2/ARE signaling and ECM metabolism-related TGF-*β*1/Smad signaling in mechanical injury-induced SUI and the relationship between these two signaling pathways.

## 2. Materials and Methods

### 2.1. Agents and Antibodies

Cell Counting Kit-8 (CCK-8) was purchased from MultiSciences Biotech Co. Ltd. (Hangzhou, China). RPMI 1640 medium was purchased from Jenom Biotech Co. Ltd. (Hangzhou, China). Fetal bovine serum (FBS) was purchased from Gibco® (Waltham, USA). Bicinchoninic acid (BCA) protein assay kit, ROS assay kit, CAT assay kit, and MDA assay kit were purchased from Beyotime Biotech Co. Ltd. (Suzhou, China). Antibodies GAPDH (ab181602), *β*-actin (ab8227), Nrf2 (ab137550), TGF-*β*1 (ab92486), COL1A1 (ab21286), COL3A1 (ab7778), 4-HNE (ab46545), 8-OHdG (ab62623), and GPx1 (ab22604) were obtained from Abcam plc. (Cambridge, UK). Antibodies elastin (sc58756), TIMP-1 (sc5538), TIMP-2 (sc5539), MMP-2 (sc10736), and MMP-9 (sc10737) were purchased from Santa Cruz Biotech Inc. (Dallas, USA). mTGF-*β*1 (5231LC) and antibodies Smad2/3 (8685), p-Smad2 (3108), and p-Smad3 (9520) were purchased from Cell Signaling Technology Inc. (Danvers, MA, USA). Antibody MnSOD (06-984) was purchased from Millipore (Billerica, USA); LY2109761 (S2704) was purchased from Selleck (Houston, USA).

### 2.2. Cell Culture and Transfection

L929 cells were purchased from Boster Biotech Co. Ltd. (product number: CX0187; Wuhan, China) and cultured in RPMI 1640 supplemented with 10% FBS and 1% antibiotics (100 IU penicillin and 100 *μ*g/mL streptomycin) in a humidified incubator at 37°C and 5% CO_2_. L929 cells were transfected using recombinant lentivirus (Genechem, Shanghai, China) with shRNA targeting Nrf2 gene (Nfe2l2, NM_010902) to establish a stably transfected L929 cell line (Nrf2 silencing). The sequence of the shRNA was 5′-CTT ACT CTC CCA GTG AAT A-3′, and the sequence of negative control (NC) shRNA is 5′- TTC TCC GAA CGT GTC ACG T -3′. Besides, a lentiviral vector (LV-Nfe2l2; Genechem, Shanghai, China) was used to establish a stably transfected L929 cell line expressing Nrf2 (Nrf2 overexpressing). Briefly, L929 cells were seeded at 1 × 10^5^ cells/well in 6-well plates 24 h prior to transfection. The L929 cells were grown to approximately 50% confluence and transfected with the recombinant lentivirus (MOI = 10) in medium with 5 *μ*g/mL polybrene for 12 h. Subsequently, the virus-containing medium was replaced with 4 mL of fresh culture medium. After 72 h, the cells were screened with medium with 4 *μ*g/mL puromycin for another 48 h. Then, measurements were conducted by q-PCR and Western blot (Figure S1 in Supplementary Material available online at https://doi.org/10.1155/2017/8524353).

### 2.3. Cell Experimental Design and Cell Treatment

**Figure 1:**
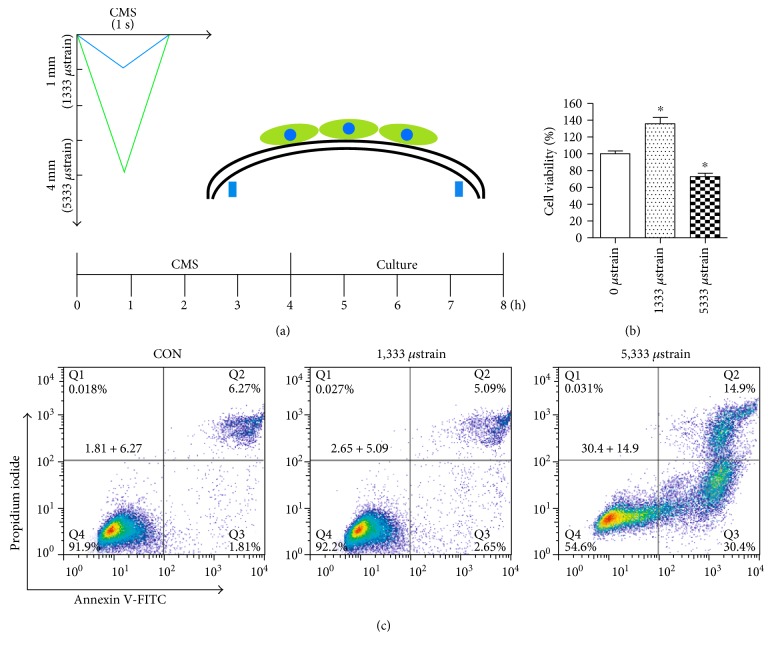
Experimental diagram and the effect of mechanical strain on L929 fibroblast. (a) Experimental diagram and the simulation diagram of mechanical strain. (b) The effect of mechanical strain on cell viabilities. (c) The effect of mechanical strain on cell apoptosis. ^∗^*P* < 0.05 compared with the control group; every experiment was repeated for 3 times. CON: control group; CMS: cyclic mechanical strain group.

In order to explore the effect of mechanical stress on L929 fibroblast, normal L929 cells underwent mechanical strain (0, 1333, and 5333 *μ*strain; 1 Hz for 4 h); then, the cells were continued to be cultured in an incubator for another 4 h, and after that, the cells were harvested for subsequent experiments (shown schematically in Figure 1(a)). In order to further confirm the involvement of TGF-*β*1/Smad signaling in mechanical injury-induced ECM remodeling, normal L929 cells were pretreated with or without mTGF-*β*1 (10 ng/mL) for 48 h; then, the experiment was continued as shown schematically in Figure 1(a) (0 and 5333 *μ*strain; 1 Hz for 4 h). In order to further confirm the involvement of Nrf2/ARE signaling in mechanical injury-induced ECM remodeling, normal L929 cells and Nrf2 overexpressing L929 cells (Lv-Nfe2l2-transfected L929 cells) were used for experiment as shown schematically in Figure 1(a) (0 and 5333 *μ*strain; 1 Hz for 4 h). In order to explore the effect of Nrf2 in mechanical injury-induced oxidative stress and damage, normal L929 cells, Nrf2 overexpressing L929 cells, and Nrf2 silencing L929 cells (Lv-shNfe2l2-transfected L929 cells) were used for experiment as shown schematically in Figure 1(a) (0 and 5333 *μ*strain, 1 Hz for 4 h). In order to explore the relationship of TGF-*β*1/Smad signaling and Nrf2/ARE signaling in mechanical injury-induced ECM remodeling, normal L929 cells, Nrf2 overexpressing L929 cells, LY2109761-pretreated Nrf2 overexpressing L929 cells, Nrf2 silencing L929 cells, and mTGF-*β*1-pretreated Nrf2 silencing L929 cells were used for experiment as shown schematically in Figure 1(a) (0 and 5333 *μ*strain, 1 Hz for 4 h). The LY2109761 pretreatment was 2 mΜ for 48 h, and mTGF-*β*1 pretreatment was 10 ng/mL for 48 h.

### 2.4. Cyclic Mechanical Strain

The L929 cells were loaded cyclic mechanical strain (CMS) according to previously described methods [19, 24] by the four-point bending device (Miracle Technology Co. Ltd., Chengdu, China); details are provided in Materials and methods in Supplementary Materials.

### 2.5. Cell Proliferation and Apoptosis Assay

After treatment according to the research design, cell proliferation and apoptosis were detected using a Cell Counting Kit-8 kit and an Annexin V-FITC/PI Apoptosis kit according to the operating manuals. Details are provided in Materials and methods in Supplementary Materials.

### 2.6. Mice and Animal Experimental Design

We performed all experiments and protocols in compliance with the institutional guidelines of the Institutional review board and received approval from the ethical committee of the Institutional Animal Care and Use Committee of Renmin Hospital of Wuhan University. We obtained wild-type (Nfe2l2^+/+^) virgin female C57BL/6 mice from the Center for Animal Experiment of Wuhan University and Nfe2l2^−/−^ mice from Jackson Laboratory (stock 017009). Thirty wild-type virgin female C57BL/6 mice (8~10 weeks old) and thirty virgin female Nfe2l2^−/−^ mice (8~10 weeks old) were randomly divided into three groups: the noninstrumented control (NC) group (WT-NC and KO-NC), sham-operated group (WT-Sham and KO-Sham), and VD group (WT-VD and KO-VD underwent vaginal distention for 1 h). There is a total of six groups, with ten mice per group. On day 6 after vaginal distention, suprapubic tube implantations were performed and leak point pressure (LPP) measurements were performed on day 7 after vaginal distention; then, all mice were sacrificed and anterior vaginal tissues were harvested for Western blot analysis and MDA assay. All mice aged from 8 to 10 weeks; the body weights of mice are shown in Table S1. Details of identification of Nfe2l2^−/−^ mice, vaginal distention, suprapubic tube implantation, and LPP measurements are provided in Materials and methods and Figure S2 in Supplementary Materials.

### 2.7. Western Blot Analysis

After treatment according to the research design, total proteins were extracted from L929 cells and vaginal walls using RIPA buffer containing PMSF and the protein expression levels were detected via Western blot analysis. Details of the Western blot analysis and information of antibodies are provided in Materials and methods in Supplementary Materials.

### 2.8. Quantitative Real-Time Polymerase Chain Reaction (Q-PCR)

Q-PCR was used to detect the mRNA expression levels of related genes. Primers were purchased from Sangon Biotech (Shanghai, China, listed in Table 1). Details of total RNA extraction, cDNA synthesis, and q-PCR are provided in Materials and methods in Supplementary Materials.

### 2.9. ROS Fluorescence Probe Assay

ROS generation in L929 cells was evaluated using the oxidant-sensing 2′,7′-dichlorofluorescein diacetate (DCFH-DA, 5 *μ*M). DCFH-DA is a nonpolar nonfluorescent dye which is converted into the polar, highly fluorescent DCF by cellular esterases in a dose-dependent manner when oxidized by intracellular ROS. The fluorescence intensity of DCFH was measured using an Olympus-BX51 fluorescence upright microscope (Olympus Corporation).

### 2.10. CAT and MDA Measurement Assay

A CAT kit and a MDA kit were used to quantify the generation of catalase (CAT) and malondialdehyde (MDA) according to the manufacturer's instructions. Details are provided in Materials and methods in Supplementary Materials.

### 2.11. Immunofluorescence

Immunofluorescence was used to detect the generation of 8-OHdG and 4-HNE and the nuclear translocation of Smad2/3. Details are provided in Materials and methods in Supplementary Materials.

### 2.12. Statistical Analysis

All statistical analyses were performed with SPSS 21.0 (IBM Corporation, Armonk, NY, USA), and data are presented here as the mean ± SD. The data were further subjected to one-way analysis of variance. Differences between two groups were determined using Dunnet's *t*-test, and multiple means were compared by Tukey's test. *P* values < 0.05 were considered statistically significant.

## 3. Results

### 3.1. The Effect of Cyclic Mechanical Strain on L929 Fibroblast

Using the experimental timeline for mechanical injury shown schematically in Figure 1(a), the viability and apoptosis of L929 cells were measured via CCK-8 assay and an Annexin V-FITC/PI Apoptosis kit after being strained for 4 h by incremental forces. As shown in Figure 1(b), the cell viability was significantly increased in the 1333 *μ*strain group and decreased in the 5333 *μ*strain group compared with that in the nonstrain control group. In addition, the cell apoptotic rate was not changed in the 1333 *μ*strain group but reduced obviously in the 5333 *μ*strain group than the nonstrain control group (Figure 1(c)). These results revealed a biphasic effect of CMS on L929 fibroblast.

### 3.2. Mechanical Trauma Induces ECM Remodeling and TIMP/MMP Imbalance on L929 Fibroblast

**Figure 2:**
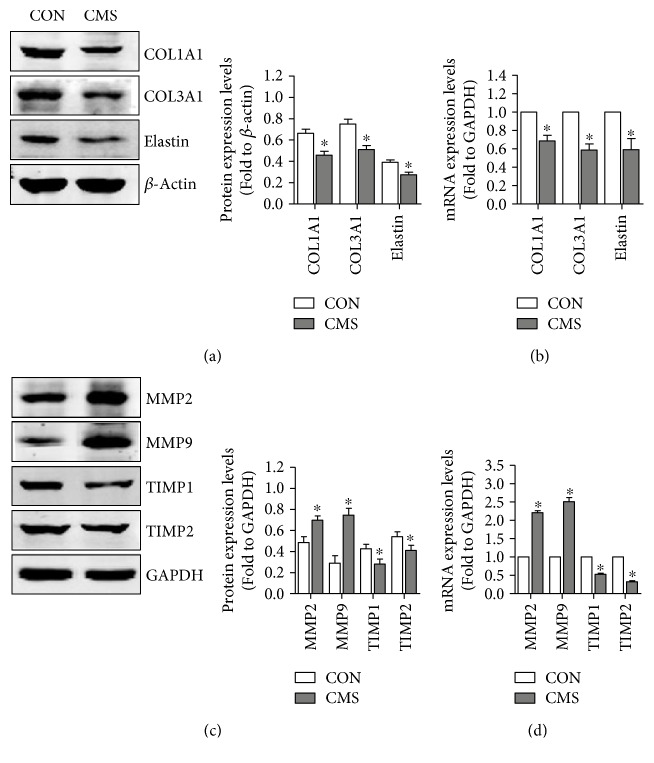
Effect of mechanical strain on ECM and MMPs/TIMPs of L929 fibroblast. (a, b) The effect of mechanical strain on protein and mRNA expression levels of ECM. (c, d) The effect of mechanical strain on protein and mRNA expression levels of MMPs and TIMPs. ^∗^*P* < 0.05 compared with the control group; every experiment was repeated for 3 times. CON: control group; CMS: cyclic mechanical strain.

ECM remodeling was one important pathomechanism of mechanical damage-induced SUI and confirmed by numerous researches. In order to investigate the effect of mechanical injury on ECM metabolism, the ECM production of L929 fibroblast was detected using Western blot and q-PCR after 5333 *μ*strain. As shown in Figures 2(a) and 2(b), both the protein and mRNA expressions of COL1A1, COL3A1, and elastin were significantly decreased in the 5333 *μ*strain group compared with those in the nonstrain control group. MMPs and their inhibitors (TIMPs) are the well-known and critical regulators of ECM metabolism. To explore the involvement of MMPs and TIMPs in mechanical injury-induced ECM remodeling, Western blot and q-PCR were used to detect the expressions of MMP-2, MMP-9, TIMP-1, and TIMP-2 in cells of two groups. Both the protein and mRNA expressions of MMP-2 and MMP-9 were upregulated obviously after 5333 *μ*strain (Figures 2(c) and 2(d)). In contrast, the protein and mRNA expressions of TIMP-1 and TIMP-2 were downregulated significantly after 5333 *μ*strain (Figures 2(c) and 2(d)).

### 3.3. Mechanical Injury Suppresses TGF-*β*1/Smad3 Signaling in L929 Fibroblast

**Figure 3:**
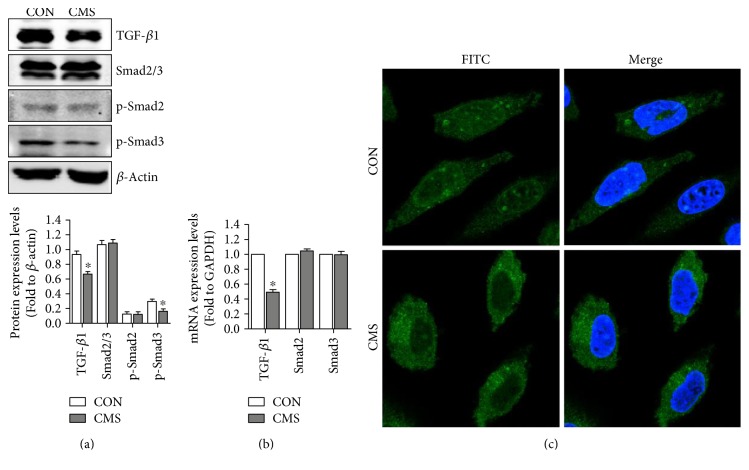
Effect of mechanical strain on TGF-*β*1/Smad signaling. (a, b) The effect of mechanical strain on protein and mRNA expression levels of TGF-*β*1/Smad signaling. (c) The effect of mechanical strain on nuclear translocation of Smad2/3. Original magnification: ×100. ^∗^*P* < 0.05 compared with the control group; every experiment was repeated for 3 times. CON: control group; CMS: cyclic mechanical strain.

TGF-*β*1/Smad signaling plays a vital role in the metabolism of ECM and has been reported to have participated in the pathological process of mechanical trauma-induced SUI. To explore the involvement of TGF-*β*1/Smad signaling in mechanical damage-induced ECM remodeling, the protein levels of TGF-*β*1/Smad signaling were determined using Western blot and q-PCR. The results showed that the protein levels of TGF-*β*1 and p-Smad3 were significantly decreased after 5333 *μ*strain than nonstrained cells, while the protein levels of Smad2/3 and p-Smad2 were not changed (Figure 3(a)). In addition, the mRNA levels of TGF-*β*1 were downregulated after stimulation with 5333 *μ*strain, whereas there were no significant changes in mRNA expressions of Smad2 and Smad3 (Figure 3(b)). Besides, mechanical injury also inhibited the nuclear translocation of Smad2/3 (Figure 3(c)).

### 3.4. Activation of TGF-*β*1/Smad Signaling Reverses Mechanical Injury-Induced ECM Remodeling of L929 Fibroblast

**Figure 4:**
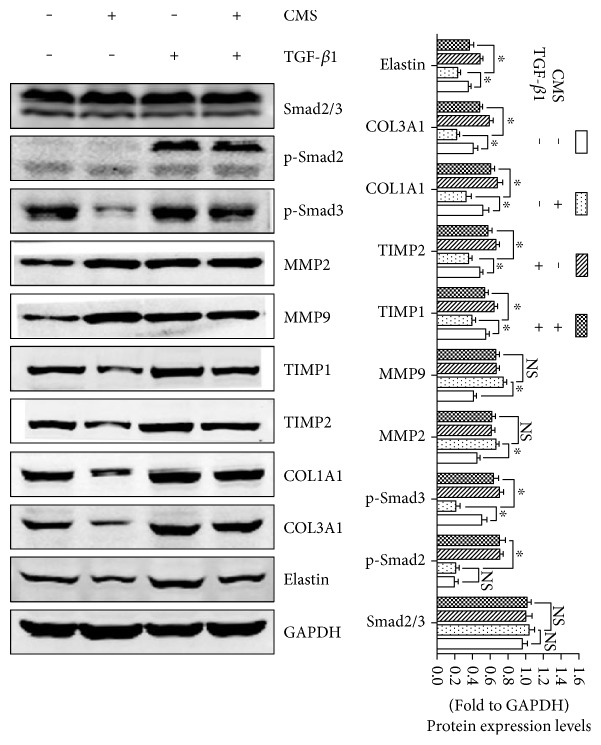
The involvement of TGF-*β*1 signaling in the process of mechanical injury-induced ECM remodeling. ^∗^*P* < 0.05; every experiment was repeated for 3 times. CON: control group; CMS: cyclic mechanical strain; TGF-*β*1: mTGF-*β*1 pretreatment.

Protein levels of TGF-*β*1/Smad3 signaling were significantly reduced in L929 cells stimulated with excessive mechanical strain. To further confirm the involvement of TGF-*β*1/Smad signaling suppression in mechanical injury-induced ECM remodeling, L929 cells were administered with mTGF-*β*1 before strain. Then, the protein levels of Smads, MMPs, TIMPs, and ECM were measured. As shown in Figure 4, the expression levels of p-Smad2 and p-Smad3 were increased obviously post-pretreatment of TGF-*β*1 both in nonstrained and in strained cells. Besides, in strained cells, the protein levels of MMP-2 and MMP-9 were not changed, whereas the expressions of TIMP-1 and TIMP-2 were significantly increased post-pretreatment of TGF-*β*1 compared with the non-pretreated group. As a consequence, the downregulation of COL1A1, COL3A1, and elastin proteins induced by mechanical damage was significantly reversed in the TGF-*β*1-pretreated group than the non-pretreated group after strain.

### 3.5. Mechanical Injury Increases ROS Levels and Oxidative Damage on L929 Fibroblast

**Figure 5:**
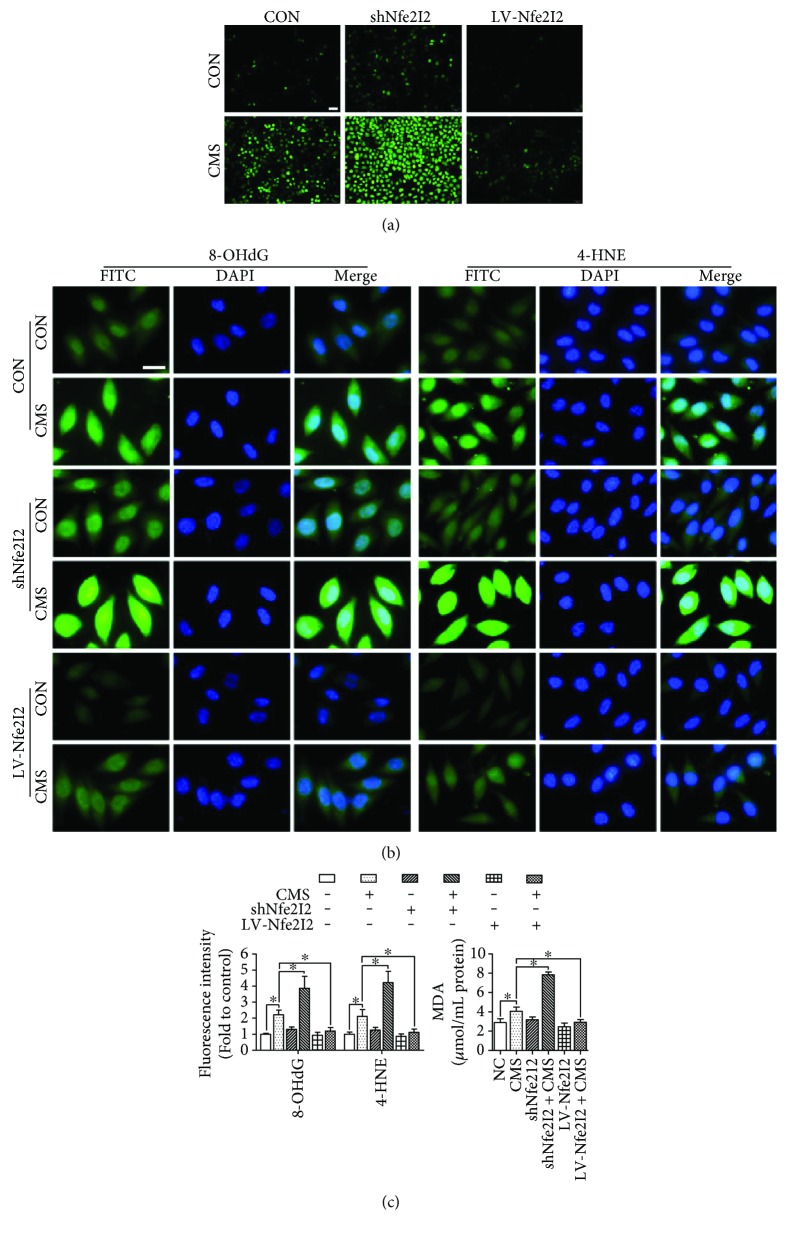
The effect of mechanical strain on ROS levels and oxidative damage on L929 cells and the involvement of Nrf2. (a) The effect of mechanical strain on ROS levels and the role of Nrf2 in this process. (b, c) The effect of mechanical strain on oxidative damage and the role of Nrf2 in this process. The scale is equal to 20 *μ*m. ^∗^*P* < 0.05; every experiment was repeated for 3 times. CON: control group; CMS: cyclic mechanical strain; shNfe2l2: Lv-shNfe2l2 transfection established Nrf2 silencing L929 cells; LV-Nfe2l2: Lv-Nfe2l2 transfection established Nrf2 overexpressing L929 cells.

The involvement of redox imbalance-induced oxidative damage of pelvic tissues in the pathomechanism of mechanical trauma-induced SUI was confirmed by numerous studies. In addition, previous researches revealed that mechanical stress-induced oxidative stress participated in fibrotic diseases. To explore the involvement of oxidative damage in mechanical injury-induced ECM remodeling, the ROS, 8-OHdG, 4-HNE, and MDA levels were determined by fluorescence probe and immunofluorescence. As shown in Figures 5(a), 5(b), and 5(c), the ROS, 8-OHdG, 4-HNE, and MDA levels were significantly increased in the 5333 *μ*strain group than in the nonstrain group.

### 3.6. Nrf2/ARE Signaling Inhibition Was Involved in Mechanical Injury-Induced Oxidative Damage on L929 Fibroblast

**Figure 6:**
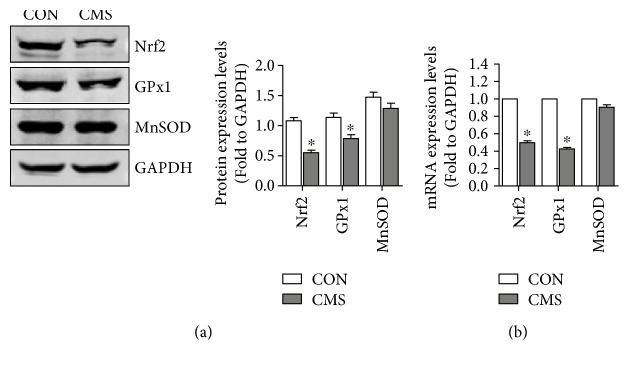
The effect of mechanical strain on Nrf2 signaling on L929 cells. (a) The effect of mechanical strain on protein expression levels of Nrf2 signaling. (b) The effect of mechanical strain on mRNA expression levels of Nrf2 signaling. ^∗^*P* < 0.05 compared with control group; every experiment was repeated for 3 times. CON: control group; CMS: cyclic mechanical strain.

Nrf2/ARE signaling, as the core member of antioxidant pathways, plays a vital role in antioxidative stress and detoxification. To investigate the involvement of Nrf2/ARE signaling in mechanical trauma-induced oxidative damage, the expression levels of Nrf2, GPx1, MnSOD, and CAT were measured. The results showed that the protein levels of Nrf2 and GPx1 (Figure 6(a)) and the concentration of CAT (Figure 5(c)) were significantly reduced after mechanical injury, and the mRNA expression levels of Nrf2, GPx1, and MnSOD were downregulated obviously in the 5333 *μ*strain group than in the nonstrain group (Figure 6(b)). In order to further confirm the participation of Nrf2/ARE signaling in mechanical injury-induced oxidative damage, Lv-Nfe2l2-transfected L929 cells and Lv-shNfe2l2-transfected L929 cells were used. As shown in Figures 5(a), 5(b), and 5(c), the overexpression of Nrf2 decreased the ROS levels and alleviated the oxidative damage induced by mechanical injury. In contrast, the silencing of Nrf2 increased the ROS levels and aggravated the oxidative damage after mechanical injury (Figures 5(a), 5(b), and 5(c)).

### 3.7. Upregulation of Nrf2/ARE Signaling Reverses Mechanical Injury-Induced and ECM Remodeling of L929 Fibroblast

**Figure 7:**
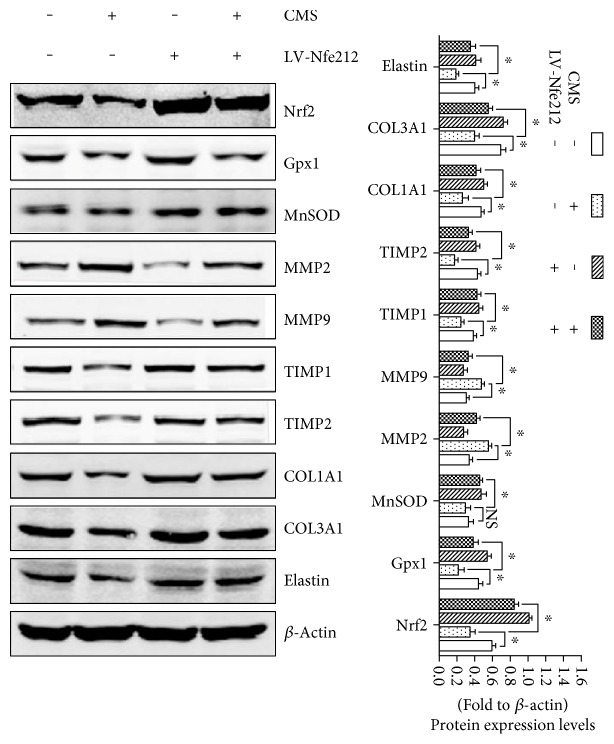
The involvement of Nrf2 signaling in the process of mechanical injury-induced ECM remodeling. ^∗^*P* < 0.05; every experiment was repeated for 3 times. CON: control group; CMS: cyclic mechanical strain; LV-Nfe2l2: Lv-Nfe2l2 transfection established Nrf2 overexpressing L929 cells.

To further confirm the involvement of Nrf2/ARE signaling inhibition in mechanical injury-induced ECM remodeling, normal L929 cells and Lv-Nfe2l2-transfected L929 cells were strained and Western blot was performed. As shown in Figure 7, the overexpression of Nrf2 significantly upregulated the protein levels of GPx1 and MnSOD and the concentration of CAT. In addition, the overexpression of Nrf2 significantly alleviated mechanical injury-induced upregulation of MMP-2 and MMP-9 and downregulation of TIMP-1 and TIMP-2. As a consequence, the overexpression of Nrf2 significantly reversed mechanical damage-induced reduction of COL1A1, COL3A1, and elastin in L929 fibroblast.

### 3.8. The Suppression of TGF-*β*1/Smad3 Signaling Was Mediated by Nrf2/ARE Signaling Inhibition in the Process of Mechanical Injury-Induced ECM Remodeling

**Figure 8:**
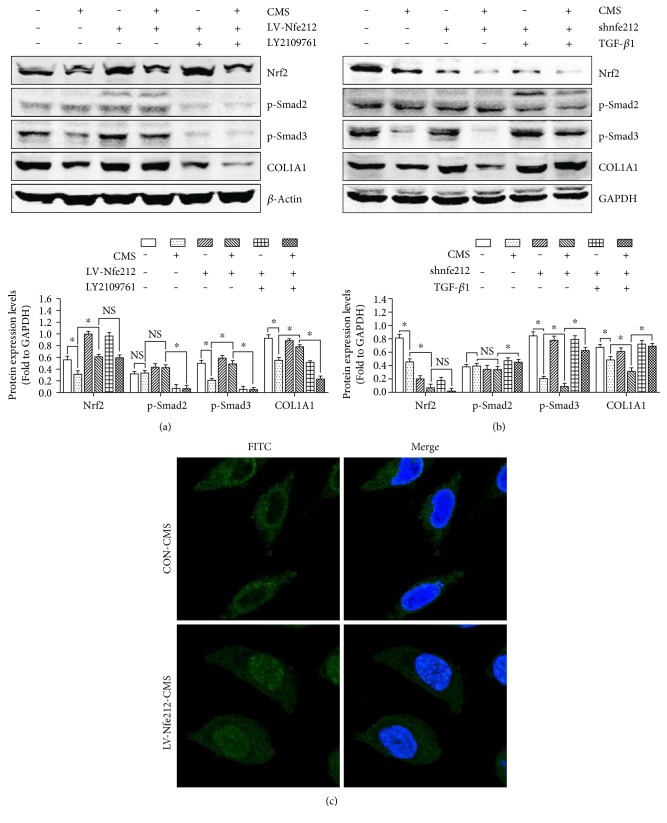
The relationship of Nrf2 signaling and TGF-*β*1 signaling in mechanical injury-induced ECM remodeling. (a) The effect of Nrf2 overexpression and LY2109761 on mechanical strain-induced TGF-*β*1 signaling inhibition. (b) The effect of Nrf2 knockdown and TGF-*β*1 on mechanical strain-induced TGF-*β*1 signaling inhibition. (c) The effect of Nrf2 overexpression on mechanical strain-induced nuclear translocation inhibition of Smad2/3. Original magnification: ×100. ^∗^*P* < 0.05; every experiment was repeated for 3 times. CON: control group; CMS: cyclic mechanical strain; shNfe2l2: Lv-shNfe2l2 transfection established Nrf2 silencing L929 cells; LV-Nfe2l2: Lv-Nfe2l2 transfection established Nrf2 overexpressing L929 cells; TGF-*β*1: mTGF-*β*1 pretreatment; LY2109761: TGF-*β*1 signaling inhibitor LY2109761 pretreatment; CON-CMS: 5333 *μ*strain mechanical strain-treated normal L929 cells; LV-Nfe2l2-CMS: 5333 *μ*strain mechanical strain-treated Nrf2 overexpressing L929 cells.

To further investigate the correlation of TGF-*β*1/Smad signaling and Nrf2/ARE signaling in the regulation of mechanical injury-induced ECM remodeling, the Lv-Nfe2l2-transfected L929 cells were administered with LY2109761 (TGF-*β*1/Smad signaling inhibitor) before CMS and the Western blot was performed. As shown in Figure 8(a), the inhibition of TGF-*β*1 signaling abrogated Nrf2 overexpression-induced reversion of mechanical injury-induced TGF-*β*1/Smad signaling inhibition and COL1A1 reduction. In order to further confirm these results, Lv-shNfe2l2-transfected L929 cells were administered with mTGF-*β*1 before CMS and Western blot was performed. The results showed that the protein levels of p-Smad2, p-Smad3, and COL1A1 in normal L929 cells were obviously higher than Nrf2 silencing cells but significantly lower than TGF-*β*1-pretreated Nrf2 silencing cells after mechanical injury (Figure 8(b)). What is more, the overexpression of Nrf2 obviously increased the nuclear translocation of Smad2/3 in strained cells than in strained normal L929 cells (Figure 8(c)). Therefore, the TGF-*β*1/Smad signaling suppression in the regulation of mechanical trauma-induced ECM remodeling may be mediated by Nrf2/ARE signaling inhibition.

### 3.9. Nrf2 Knockout Aggravates Mechanical Trauma-Induced LPP Reduction, ECM Remodeling, Oxidative Damage, and TGF-*β*1/Smad3 Signaling Suppression in Mice

**Figure 9:**
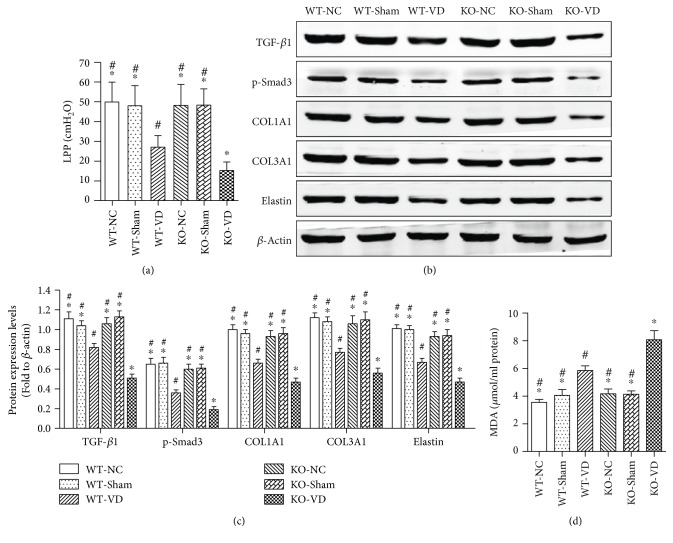
In vivo confirmation on vaginal distension-induced SUI mice model. (a) LPPs of mice in six groups. (b, c) Protein expression levels of TGF-*β*1/Smad3 signaling and ECM in anterior vaginal walls of mice in six groups. (d) MDA concentrations in anterior vaginal walls of mice in six groups. ^∗^*P* < 0.05 compared with the WT-VD group. ^#^*P* < 0.05 compared with the KO-VD group; every experiment was repeated for 3 times. WT-NC: noninstrumented control group of wild-type mice; WT-Sham: sham-operated group of wild-type mice; WT-VD: VD group of wild-type mice; KO-NC: noninstrumented control group of Nrf2 knockout mice; KO-Sham: sham-operated group of Nrf2 knockout mice; KO-VD: VD group of Nrf2 knockout mice.

To confirm the results of above in vitro researches, an in vivo SUI model was used in our study. The results showed that there were no significant differences among the LPPs of mice in the WT-NC, WT-Sham, KO-NC, and KO-Sham groups (Figure 9(a)). But the LPPs of mice in the WT-VD group were significantly lower than those in the WT-NC, WT-Sham, KO-NC, and KO-Sham groups, but significantly higher than those in the KO-VD group (Figure 9(a)). Similarly, the protein levels of COL1A1, COL3A1, elastin, TGF-*β*1, and p-Smad3 in anterior vaginal walls of mice in the WT-VD group were significantly lower than those in the WT-NC, WT-Sham, KO-NC, and KO-Sham groups, but significantly higher than those in the KO-VD group (Figures 9(b) and 9(c)). Besides, the generation of MDA in anterior vaginal walls of mice was increased in the WT-VD group than that in the WT-NC, WT-Sham, KO-NC, and KO-Sham groups, but lower than that in the KO-VD group (Figure 9(d)). And there were no significant differences among the levels of COL1A1, COL3A1, elastin, TGF-*β*1, p-Smad3, and MDA in anterior vaginal walls of mice in the WT-NC, WT-Sham, KO-NC, and KO-Sham groups (Figures 9(b), 9(c), and 9(d)).

## 4. Discussion

Vaginal delivery is among the recognized risk factors for SUI. The mechanisms of pregnancy- and delivery-related SUI are not very clear. Vaginal delivery can injure the ECM, muscle, and nerve responsible for maintaining continence [25–27]. In addition, the stretch injury may cause alteration of the composition and function of the lower urinary tract tissues. In this study, we explored the potential mechanisms of mechanical trauma-induced SUI in vitro, and the results showed that excessive mechanical stress can induce the inhibition of Nrf2/ARE signaling and then suppress the antioxidant ability of cells. As a result, excessive accumulation of ROS induces oxidative damage of DNAs, lipids, and proteins and suppression of TGF-*β*1/Smad signaling could further result in remodeling of ECM.

To investigate the effect of cyclic mechanical strain on fibroblast, L929 cells underwent 0, 1333, and 5333 *μ*strain for 4 h in 1 Hz. The results showed that suitable mechanical strain could increase the cell viability, while excessive force could reduce the cell viability. Similarly, the cell apoptosis assay indicated that the apoptosis rate of cells has not changed in the smaller mechanical strain group but significantly decreased in the excessive mechanical strain group compared with the nonstrain control group. Reasons for this phenomenon might be the growth-promoting effect of physiological levels of ROS and suitable mechanical strain-induced activation of latent TGF-*β*1 [12]. Besides, we investigate the metabolic changes of ECM after excessive mechanical strain on L929 cells. The protein and mRNA expression levels of COL1A1, COL3A1, and elastin were significantly decreased after 5333 *μ*strain.

MMPs are zinc-dependent proteases that play important roles in ECM degradation in many tissues. TIMPs are the inhibitor of MMPs. Moreover, the results of many studies have indicated that MMP-2 expression is significantly higher in VSMCs after stretching [22]. In this study, our data revealed that both the protein and mRNA expression levels of MMP-2 and MMP-9 were significantly increased after CMS; in contrast, the protein and mRNA expression levels of TIMP-1 and TIMP-2 were decreased compared with nonstrain control cells. This might be an important middleman of mechanical stress-induced ECM remodeling.

TGF-*β*1/Smad signaling plays a vital role in the metabolism of ECM and has been reported to have been involved in the pathological process of mechanical trauma-induced SUI [28, 29], while the effect of excessive mechanical strain on TGF-*β*1/Smad signaling and the underlying mechanism was still unclear. In order to further elucidate the mechanism of mechanical injury-induced ECM remodeling and the role of TGF-*β*1/Smad signaling in this process, we detected the expressions of TGF-*β*1/Smad signaling-related factors in both 5333 *μ*strain cells and nonstrain control cells. Our results showed that 5333 *μ*strain significantly suppressed the activation of TGF-*β*1/Smad3 signaling and the nuclear translocation of Smad2/3. And the pretreatment of mTGF-*β*1 significantly activated mechanical injury-induced suppression of TGF-*β*1/Smad3 signaling and reversed the imbalance of MMPs/TIMPs and the reduction of ECM proteins. Therefore, TGF-*β*1/Smad signaling inhibition may be a vital intermediate link of mechanical injury-induced ECM remodeling. However, many previous studies indicated that latent TGF-*β*1 in ECM would be activated by suitable mechanical strain and presents a growth-promoting effect.

ROS accumulation is a result of mechanical stress, a suitable mechanical strain that would induce a raise of ROS levels in physiological range, while the excessive mechanical stress would cause an excessive ROS accumulation and result in oxidative damage. Previous study suggested that exogenous ROS had an inhibitory effect on TGF-*β*1 signaling which induced a reduction of ECM production [24, 30]. Our data revealed that the ROS levels and the production of 8-OHdG, 4-HNE, and MDA, the biomarkers of oxidative damage, were significantly increased in the mechanical injury group than in the nonstrain group. In order to explore the cause of increase of ROS, we detected the expression levels of Nrf2 signaling-related factors which were the major antioxidant pathways. The results showed that excessive mechanical stress suppressed Nrf2 signaling via the downregulation of Nrf2, GPx1, and CAT expressions. Besides, the overexpressing of Nrf2 significantly alleviated mechanical injury-induced ROS accumulation and oxidative damage and reversed the imbalance of MMPs/TIMPs and reduction of ECM production. Therefore, we deduced that Nrf2 signaling inhibition and excessive ROS-induced oxidative damage may be a vital intermediate link of mechanical injury-induced ECM remodeling.

Based on the results above, to explore whether excessive ROS accumulation plays a vital role in mechanical injury-induced TGF-*β*1/Smad signaling inhibition, we validated the interrelation of TGF-*β*1/Smad signaling and Nrf2/ARE signaling. Our data showed that TGF-*β*1/Smad signaling suppression in the regulation of mechanical trauma-induced ECM remodeling may be mediated by Nrf2/ARE signaling inhibition. Hence, we deduced that the excessive mechanical strain could inhibit the activation of Nrf2/ARE signaling and increase the production of ROS in L929 fibroblast; then, the excessive levels of ROS would suppress the mechanical stress-induced activation of latent TGF-*β*1 in ECM and inhibit the nuclear translocation of Smad2/3, break the balance of MMPs/TIMPs, and eventually result in ECM remodeling. Although excessive mechanical strain could suppress the activation of Nrf2 signaling, it may be not the only cause of excessive ROS accumulation. A previous study indicated that mechanical stretch could raise the ROS production via the mitochondria damage, calcium influx, NADPH oxidase, and integrin signaling [22, 31].

In order to further confirm the results of in vitro researches, a vaginal distention-induced in vivo SUI model on wild-type and Nrf2-knockout mice was used. As described in our previous study, we detected the LPPs of mice and protein expression levels of anterior vaginal walls on day 7 after VD [32]. The results showed that Nrf2 knockout aggravated mechanical trauma-induced LPP reduction and exacerbated mechanical trauma-induced ECM remodeling, oxidative damage, and TGF-*β*1/Smad3 signaling suppression in anterior vaginal walls of mice. The vesical neck and urethra are attached to the anterior vaginal wall, which has fascial connections to the levator ani muscles through the arcus tendineus fasciae pelvis [4]. Collagen, elastin, and muscular tissue enable the vaginal wall to have enough strength and toughness to maintain the normal anatomic position and function. Hence, childbirth injury could induce oxidative damage of pelvic tissues via Nrf2/ARE signaling inhibition-mediated TGF-*β*1/Smad3 suppression and increases ECM catabolism and decreases ECM synthesis; then, the urethra will lose the hammock-like support and result in SUI.

In conclusion, our results suggest that mechanical strain-induced ECM remodeling of L929 fibroblasts might be associated with Nrf2/ARE signaling suppression mediating TGF-*β*1/Smad3 signaling inhibition, which might be the pathomechanism of mechanical trauma-induced SUI. And it might be an effective target for the treatment and prevention of SUI. However, childbirth injury induced varying degrees of damage to connective tissues, muscle tissues, and nerve tissues that may all be involved in the pathogenesis of SUI. In this study, we just studied the possible mechanism of mechanical trauma-induced extracellular matrix remodeling on fibroblasts in vitro and on vaginal walls in vivo. Further in vitro and in vivo researches about mechanical injury of muscle and nerve cells and tissues are needed to confirm the result of this research. Meanwhile, antioxidant therapy might be a possible new research orientation for the study of the prevention and treatment of SUI.

## Supplementary Material

Supplementary Material 1: Materials and methods. Supplementary Figures. Fig. S1: Validation of cell transfection. Fig. S2: Validation of Nrf2 knockout mice (Nfe2l2^−/−^). Supplementary Tables. Table S1: The body weights of mice in six groups ($\bar{x}$±*S*).

## Figures and Tables

**Table 1:** The primers for the quantitative real-time polymerase chain reaction.

Gene name	Gene ID	Primer sequence (5′-3′)	Amplicon size (bp)
Collagen I (A1)	NM_007742.3	F: AAGAAGCACGTCTGGTTTGGAG	175
		R: GGTCCATGTAGGCTACGCTGTT	
Collagen III (A1)	NM_009930	F: GTGGCAATGTAAAGAAGTCTCTGAAG	191
		R: GGGTGCGATATCTATGATGGGTAG	
Elastin	NM_007925.4	F: CTGGTGTTGGTCTTCCAGGT	112
		R: GCTTTGACTCCTGTGCCAGT	
Nrf2	NM_010902.3	F: CTGGCTGATACTACCGCTGTTC	208
		R: AGGTGGGATTTGAGTCTAAGGAG	
GPx1	NM_001329527.1	F: CAATGTCGTTGCGGCACACC	135
		R: CCTCAAGTACGTCCGACCTG	
MnSOD	NM_013671.3	F: AACTCAGGTCGCTCTTCAGC	121
		R: CTCCAGCAACTCTCCTTTGG	
TGF-β1	NM_011577	F: CTAATGGTGGACCGCAACAAC	99
		R: CACTGCTTCCCGAATGTCTGA	
Smad2	NM_001252481.1	F: GCTCAAGGCAATCGAAAACT	121
		R:CCGAGGCACTAAGACTGGAG	
Smad3	NM_016769.4	F: CACAGCCACCATGAATTACG	120
		R:TGGAGGTAGAACTGGCGTCT	
MMP-2	NM_008610.3	F: ACCCAGATGTGGCCAACTAC	121
		R: AAAGCATCATCCACGGTTTC	
MMP-9	NM_013599.4	F: AGACGACATAGACGGCATCC	116
		R: TGGGACACATAGTGGGAGGT	
TIMP-1	NM_001044384.1	F: CCCAGAAATCAACGAGACCACC	241
		R: ACGCCAGGGAACCAAGAAGC	
TIMP-2	NM_011594.3	F: GACACGCTTAGCATCACCCA	127
		R: CCATCCAGAGGCACTCATCC	
GAPDH	NM_008084.2	F: AGGAGCGAGACCCCACTAACA	247
		R: AGGGGGGCTAAGCAGTTGGT	

## Abbreviation

TGF-*β*1: Transforming growth factor-*β*1
mTGF-*β*1: Recombinant mouse transforming growth factor-*β*1
Nrf2: Nuclear factor-erythroid 2 p45-related factor 2
ARE: Antioxidant response element
GPx1: Glutathione peroxidase-1
MnSOD: Manganese superoxide dismutase
CAT: Catalase
8-OHdG: 8-Hydroxy-2′-deoxyguanosine
4-HNE: 4-Hydroxynonenal
MDA: Malondialdehyde
MMP: Matrix metalloproteinase
TIMP: tissue inhibitors of metalloproteinase
ROS: Reactive oxygen species
Lv-shNfe2l2: Lentivirus-based vector with shRNA target to Nfe2l2
Lv-Nfe2l2: Lentivirus vector used to establish a stably transfected L929 cell line overexpressing Nrf2
VD: Vaginal distention
WT: Wild-type
KO: Knockout
LPP: Leak point pressure.
