# Supplementary material for: Mechanism of Mechanical Trauma-Induced Extracellular Matrix Remodeling of Fibroblasts in Association with Nrf2/ARE Signaling Suppression Mediating TGF-β1/Smad3 Signaling Inhibition
Source: Oxid Med Cell Longev. 2017 Oct 3;2017:8524353. doi: 10.1155/2017/8524353 (PMC5646330; doi:10.1155/2017/8524353)
Supplement: Supplementary file 1 — Supplementary Material 1: Materials and methods. Supplementary Figures. Fig. S1: Validation of cell transfection. Fig. S2: Validation of Nrf2 knockout mice (Nfe2l2−/−). Supplementary Tables. Table S1: The body weights of mice in six groups (x¯±S). [file 8524353.f1.pdf]

**Supplementary Materials**

**S1 Materials and methods**

**Cyclic mechanical strain** The L929 cells were loaded CMS by the four-point bending device(Miracle Technology Co., Ltd., Chengdu, China), which was divided into three parts: Mechanical power systems, host computer and strain-loading dish. The cell suspension(1.5 ml, containing about $2\times10^5$ cells) were seeded onto the culture plate uniformly, and placed in incubator with condition of 5% $CO_2$ and 37℃ for 48 h. The culture plate containing cells were transferred to the strain-loading dish for mechanical strain until cells were adherent to the plate and reached ninety percent confluence. Strain parameters were set to a frequency of 1 Hz for 4 h, and the cells were subjected to strains of 0 mm (control group samples), 1 mm (1333 µ) and 4 mm (5333 µ) according the experimental design.

**Cell-proliferation and apoptosis assay** Cells were seeded onto plates at a density of $2\times10^5$ cells per plate and then incubated for 48 hours. Then, cells were treated with CMS. After that, Cell viability(equal amount of cells) was measured using the Cell Counting Kit-8 method according to the manufacturer's protocol. At last, the optical density at 450 nm was detected by using a PerkinElmer Victor3 1420 Multilabel Counter (PerkinElmer, Inc., Waltham, MA, USA). Optical density values represent cell viability. The apoptosis of cells was quantified with Flow Cytometry Assay, after CMS, Cells were stained with Annexin V and PI using an Annexin V-FITC/PI Apoptosis kit according to the manufacturer's instructions. Briefly, L929 cells were digested from plates and washed twice with cold phosphate-buffered saline (PBS) and resuspended in Annexin V-FITC binding buffer. Annexin V-FITC was then added and mixed gently, incubated for 15 min at 4℃ in the dark. Then, PI staining solution was then added and incubated for 5 min at 4℃ in the dark.. The cells were kept on ice in the dark and immediately analyzed by FACSCalibur system (BD, Franklin Lakes, NJ, USA)

**Vaginal distention** Mice in the VD groups were underwent vaginal distention after anesthetized with urethane (1g/kg, i.p.). After lubricated with paraffin oil, modified 6-Fr. Foley catheter was inserted into the vagina and secured to the vaginal introitus with a 5/0 silk suture. Then 0.3 ml distilled water was infused into the balloon to distend the vagina. Each balloon's diameter was measured before VD using a Vernier caliper. After 1 h, the balloon was deflated and removed, and the mouse was allowed to awaken from the anesthesia spontaneously. The NC groups have not underwent vaginal dilation and sham groups just inserted modified 6-Fr. Foley catheter into the vagina and secured with a 5/0 silk suture without distilled water infusion.

**Suprapubic Tube Implantation and LPP Measurement** One day before LPP measurement, a epidural catheter was implanted in the bladder under urethane (1 g/kg, i.p.) anesthesia. In the LPP performing day, the mice were anesthetized with urethane (1 g/kg, i.p.). Then the bladder catheter was connected to both a micro syringe pump and a pressure transducer of urinary dynamics detector (Nidoc970C, Weixin Medical of China) through a T-branch pipe. Pressure and force transducer signals were amplified and digitized for computer data collection. The bladder was then filled with

room-temperature saline at 1 ml/hr through the bladder catheter. When half bladder capacity was reached, gentle pressure with one finger was applied to the mouse's abdomen Pressure was gently increased until urine leaked, at which time the externally applied pressure was rapidly removed. The peak bladder pressure was taken as the LPP. Voids could be easily distinguished from leaks. If a mouse voided, the bladder was refilled and the process was repeated. At least five LPPs were obtained on each animal and the mean LPP was calculated.

**Western blot analysis** Total proteins were extracted from L929 cells and vaginal walls using RIPA buffer containing PMSF. To measure protein concentrations, a BCA assay kit (Beyotime, China) was used according to the manufacturer's instructions. After adding protein loading buffer (200 mM of DTT, 40 mM of Tris/HCl, 40% glycerol, 4% SDS; pH 6.8, 0.032% Bromophenol blue) and denaturing at 95°C for 5 minutes, 30 μg of the total protein was separated from these samples by 10% SDS–polyacrylamide gel electrophoresis (PAGE), and then transferred onto activated PVDF membranes. After blocking in 5% skimmed milk at 37℃ for 1 hour, the membranes were blotted with appropriate primary antibodies at 4℃ overnight, followed by fluorescence-labeled secondary antibodies (IRDye700 and IRDye800, goat antimouse/rabbit,1:10000) for 1 hour at 37℃. The primary antibodies information as follows: anti-Nrf2(1:500), anti-GPx1(1:500), anti-MnSOD (1:500), anti-TGF-β1(1:250), anti-Smad2/3(1:1000), anti-p-Smad2(1:250), anti-p-Smad3(1:250), anti-COL1A1(1:2000), anti-COL3A1(1:5000) anti-elastin (1:200), anti-MMP2(1:200), anti-MMP-9(1:200), anti-TIMP-1(1:1000), anti-TIMP-2(1:1000), anti-GAPDH(1:2000) and anti-β-actin(1:5000). Signals were detected with an Odyssey infrared imaging system (LI-COR Biosci-ences, Lincoln, NE, USA).

**Quantitative real-time polymerase chain reaction(q-PCR)** Primers were purchased from Sangon Biotech(Shanghai, China). Total RNA was extracted using RNAiso Plus (TaKaRa Biotech, Dalian, China), and first-strand complementary (c)DNA was synthesized using a PrimeScriptTM RT regent Kit (TaKaRa Biotech, Dalian, China). Quantitative real-time PCR was conducted using SYBR® Premix Ex TaqTM II Kit(TaKaRa Biotech, Dalian, China). SYBR green real-time PCR mix for PCR containing 7.5 μM each of forward and reverse primers. The reaction conditions were as follows: predenaturation at 95°C for 30 seconds; 40 cycles of 95°C for 5 seconds and 60°C for 34 seconds; and a final extension stage of 95°C for 15 seconds, 60°C for 1 minute and 95°C for 15 seconds. β-actin was used as the reference gene. Relative quantification of gene expression for both target and reference genes was performed by the $2^{-\Delta\Delta Ct}$ method and based on Ct values. Real-time PCR analysis results are presented as the mean ± standard deviation (SD) of fold-change in expression.

**CAT and MDA measurement assay** After total protein extraction from L929 cells and vaginal walls using RIPA buffer containing PMSF. A BCA assay kit was used according to the manufacturer's instructions to detect the protein concentrations. Then, the cell extract were collected for measurements of human catalase activity by a catalase analysis kit following the manufacturer's

instructions. Briefly, samples were treated with excess hydrogen peroxide for decomposition by catalase for an exact time, and the remaining hydrogen peroxide coupled with a substrate was treated with peroxidase to generate a red product, *N*-4-antipyryl-3-chloro-5-sulfonate-*p*-benzoquinonemonoimine, which absorbs maximally at 520 nm. Catalase activity was thus determined by measuring the decomposition of hydrogen peroxide spectrophotometrically. For lipid peroxidation assay, we used a malondialdehyde(MDA) analysis kit to quantify the generation of MDA according to the manufacturer's protocol. In brief, cells were harvested by trypsinization and cellular extracts were prepared by sonication in ice-cold buffer (50 mM Tris-HCl, pH 7.5, 5 mM EDTA, and 1 mM DTT). After sonication, lysed cells were centrifuged at 10,000 × *g* for 20 min to remove debris. The supernatant was subjected to the measurement of MDA levels and the protein contents. MDA levels were then normalized to milligram protein. We used the same procedure to lyse the cells and determine the protein contents in the following assays unless otherwise indicated.

**Immunofluorescence** After treatment, cells were washed twice with PBS and fixed in 4% paraformaldehyde for 15 minutes, and then permeabilized using 0.3% Triton X-100 buffer for 5 minutes at room temperature. Afterward, the cells were extensively washed thrice with PBS and blocked with 5% goat serum in PBS for 1 h at room temperature, then incubated with the primary antibodies at 4℃ overnight. The polyclonal antibodies were used: anti-8-OHdG (1:200), anti-4-HNE (1:200) and anti-Smad2/3(1:200). To visualize the primary antibodies, the cells were stained with fluorescein isothiocyanate or cyanine dye-labeled secondary antibodies (1:100). Stained cells were mounted using the antiquenching fluorescence mounting medium (Wuhan Good Biotech, Wuhan, China) and viewed using an Olympus-BX51 fluorescence upright microscope (Olympus Corporation). Quantitation of immunofluorescence staining was carried out on coded cell coverslips as the integrated option density value.

**S2 Figures:**

**Fig S1:**

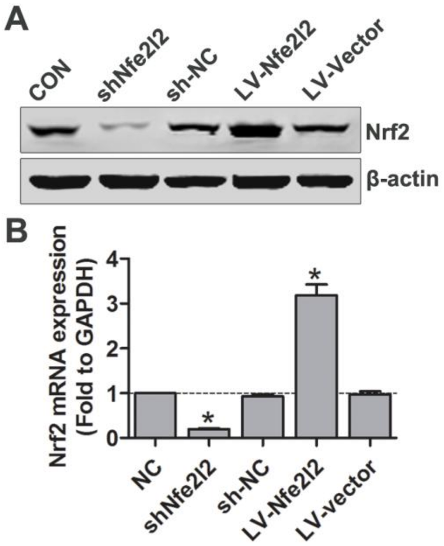


**Fig. S1 Validation of cell transfection**. **A**. Nrf2 protein expression levels of L929 cells; **B**. mRNA expression levels of L929 cells. *represents P＜0.05, every experiment was repeated for 3 times. (CON: normal L929 cells; shNfe2l2: Lv-shNfe2l2 transfection established Nrf2 silencing L929 cells; sh-NC: negative control shRNA transfected L929 cells; LV-Nfe2l2: Lv-Nfe2l2 transfection established Nrf2 over-expressing L929 cells; LV-Vector: empty vector transfected L929 cells)

**Fig. S2**:

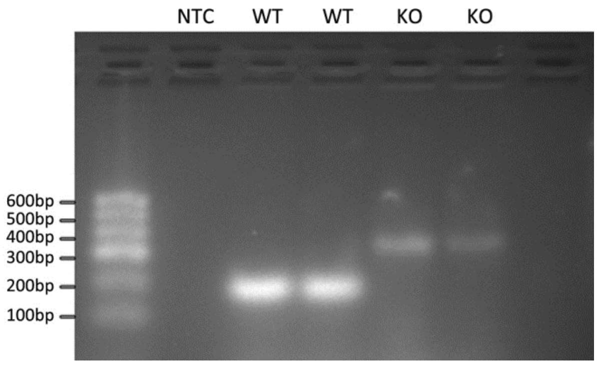


**Fig. S2 Validation of Nrf2 knockout mice(Nfe2l2$^{-/-}$)** Mice were validated via standard PCR according to the instruction of Jackson Laboratory (https://www2.jax.org/protocolsdb/f?p=116:5:0::NO:5:P5_MASTER_PROTOCOL_ID,P5_JRS_CODE:7474,017009) ; Mutant = ~400 bp, Heterozygote = ~400 bp and 262 bp, Wild type = 262 bp; (WT: wild-type mice; KO: Nrf2 knockout mice; NTC: PCR reaction mixture control without DNA template)

**S3 Tables:**

**Table S1:**

Table S1: The body weights of mice in six groups( $\overline{x}$ ±*S*)

| Group | | Weight(g) | F | *P* |
|---|---|---|---|---|
| WT | NC | 17.20±0.48 | 0.37 | 0.86 |
| | Sham | 17.01±0.49 | | |
| | VD | 17.14±0.44 | | |
| KO | NC | 17.04±0.44 | | |
| | Sham | 16.99±0.46 | | |
| | VD | 17.17±0.47 | | |
